# Tartary Buckwheat Bran and Fructus Aurantii Combination (TBB‐FA): A Promising Therapeutic Approach for Functional Dyspepsia via Modulation of Gut Microbiota, Short‐Chain Fatty Acids and Purine Signaling Pathway

**DOI:** 10.1002/fsn3.4695

**Published:** 2025-01-12

**Authors:** Di Wang, Xiaobo Zeng, Xinge Wang, Wen Wen, Ping Wang, Shijun Xu

**Affiliations:** ^1^ Southwest State Key Laboratory of Traditional Chinese Medicine Resources, School of Pharmacy Chengdu University of Traditional Chinese Medicine Chengdu China

**Keywords:** fructus aurantii, functional dyspepsia, metabolomics, purinergic signaling, short‐chain fatty acids, Tartary buckwheat bran

## Abstract

This study evaluates the therapeutic impact of Fructus aurantii (FA) stir‐baked with tartary buckwheat bran (TBB) on functional dyspepsia (FD), employing a reserpine at the dose of 5 mg/kg to rats. FA, a traditional Chinese herbal medicine, is processed with TBB to enhance its gastrointestinal motility benefits. The study's objectives were to assess the impact of this preparation on intestinal flora, SCFA levels, and metabolomic profiles in FD. Rats were divided into groups receiving different treatments, with the TBB‐FA group showing a 7.15–33.2 times increase in fecal SCFA levels, specifically propionate and butyrate, compared to the Fructus aurantii (FA) stir‐baked with wheat bran (WB) group (WB‐FA) (*p* < 0.05). Metabolomics identified 23 serum and 11 intestinal mucosal biomarkers associated with FD, predominantly linked to the purine metabolic pathway. Results indicated a significant positive correlation (*r* ≥ 0.7) between the abundance of Bacteroides and the expression of propionate and isobutyrate in fecal samples post‐TBB‐FA treatment. This suggests that TBB‐FA may enhance beneficial gut bacteria and SCFA production, potentially modulating the purinergic signaling pathway, which is implicated in gastrointestinal motility. In conclusion, the study demonstrates that TBB‐FA could be a promising therapeutic approach for FD by improving gut microbiota and SCFA levels and highlights the purinergic signaling pathway as a novel target for treatment. The findings pave the way for further research into the integration of traditional Chinese medicine and modern therapeutic strategies for FD.

## Graphical abstract

Fructus aurantii, stir‐baked with Tartary buckwheat bran, was employed as a treatment for functional dyspepsia (FD). The potential of this combination to replace or supplement wheat bran was thoroughly assessed. The purinergic signaling system holds potential as a therapeutic target for gastrointestinal disorders, thereby facilitating the development of innovative pharmaceuticals for the clinical management of FD.

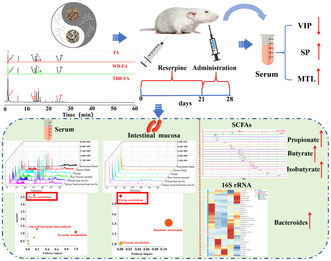

## 1 Introduction

Functional dyspepsia (FD) is a prevalent functional gastrointestinal disorder in clinical practice, yet its etiology remains incompletely understood. The prevailing view attributes FD primarily to gastrointestinal motility disorders, with current clinical management lacking specific therapeutic interventions (Ford et al. 2020). Consequently, there is a pressing need for novel breakthroughs to investigate alternative treatment mechanisms. Recent research has highlighted the significance of intestinal flora imbalance in the pathogenesis of FD, suggesting that modulating intestinal flora levels and their metabolites, particularly short‐chain fatty acids, may hold potential (Xu et al. 2020).

Fructus aurantii (FA), a frequently utilized Chinese herbal medicine in FD treatment, is known for its ability to regulate qi, alleviate abdominal bloating, and enhance gastrointestinal peristalsis (Qiao et al. 2021). Traditional Chinese medical practitioners historically favored the use of processed FA Xin powder over raw forms due to its milder effects. Processing methods typically involve stir‐baking with wheat bran (WB), a practice believed to mitigate dryness and enhance spleen‐invigorating and digestion‐relieving properties (Li et al. 2021; Zhu et al. 2020). Notably, tartary buckwheat bran (TBB) shares similarities with WB in taste and dietary fiber content, both of which are associated with improved gastrointestinal motility (He et al. 2022). Previous studies exploring excipient substitution demonstrated that Fructus aurantii (FA) stir‐baked with tartary buckwheat bran (TBB‐FA) significantly enhanced the presence of quality markers related to “broadness and distension” in fructus citri, suggesting the potential for TBB to replace WB as a resourceful and beneficial alternative (Noda et al. 2023). Moreover, ancient Chinese medical texts have documented the beneficial effects of buckwheat in promoting intestinal health and reducing accumulation, aligning with the therapeutic properties of FA in regulating qi and alleviating abdominal distension (Liu et al. 2021). Consequently, the incorporation of TBB as an adjunct material FA warrants exploration in this study.

The pharmacological treatment of FD can involve drugs that act directly on the disease target or influence metabolic pathways by modulating intestinal flora and short‐chain fatty acids (SCFAs) levels (Dal Ben et al. 2020). Studies by (Yang et al. 2024; Zhang et al. 2024) demonstrated that certain drug administrations in FD animal models led to increased abundance of specific microflora and SCFAs levels, resulting in improved gastrointestinal motility and symptom relief. Specifically, lactis BL‐99 was found to enhance SCFA production and alter gut microbiota composition, potentially impacting gastrin production and alleviating FD symptoms. This research delves into the therapeutic mechanism of TBB‐FA in FD treatment, focusing on the interplay between intestinal microbiota, SCFAs, and metabolomics. The objective of this research was to assess the viability of substituting wheat bran with tartary wheat bran in the frying process of Fructus aurantii in FD treatment, offering novel insights for clinical practice, traditional Chinese medicine processing, and sustainable utilization of buckwheat resources.

## 2 Material and Methods

### 2.1 Materials and Reagents

FA, after undergoing the removal of impurities and the nucleus, was sourced from Sichuan and acquired from Sichuan Qiyuan Pharmaceutical Co. LTD. This botanical material was authenticated as the desiccated unripe fruit of *C. aurantium* , a member of the Rutaceae family, by Professor Chen Hongping from Chengdu University of Traditional Chinese Medicine. The TBB originated from Miqiao No.1, cultivated in Liangshan, Sichuan Province. High‐performance liquid chromatography (HPLC) grade acetonitrile and formic acid were procured from Fisher Scientific in the USA. Distilled water was obtained from Watsons Corporation in China. Various acids including formic acid (C1), acetic acid (C2), propionic acid (C3), butyric acid (C4), isobutyric acid (C4; 2‐methylpropionic acid), 2‐methylbutyric acid (C5), caproic acid (C6; hexanoic acid), 2‐ethylbutyric acid (C6), and 2‐methylvaleric acid (C6; 2‐methylpentanoic acid) were purchased from Sigma‐Aldrich in St. Louis, MO, USA. Additionally, AABD‐SH (4‐acetylamino‐7‐mercapto‐2,3‐benzooxadiazole), TPP (triphenylphosphine), and DPDS (2,2′‐dipyridine disulfide) were acquired from Shanghai Maclin Biochemical Technology Co. LTD.

### 2.2 Production of FA From Various Processed Forms

The processing techniques for FA stir‐baked with bran, as delineated in the 2020 edition of the Chinese Pharmacopeia, were adhered to in this study. This involved selecting 500 g of FA decoction pieces along with 50 g each of WB and TBB. The process entailed heating a frying pan, adding the bran until smoking, then introducing the FA decoction pieces for quick stir‐baking until the surface achieved a yellow or dark yellow hue. Subsequently, the bran was sieved out and cooled, resulting in the production of WB and TBB fried FA. Separately, 150 g of the two types of FA were placed in a casserole along with 2250 mL of distilled water for a 30‐min soaking period. This mixture was decocted and extracted thrice for 15 min each time. The resulting extraction solution was filtered and concentrated to a density of 1.2 g/mL, and the quantity of raw drug was duly recorded.

### 2.3 Animal

Adult male Sprague–Dawley rats of specific pathogen‐free (SPF) grade, weighing between 220 and 240 g, were procured from Spefer Biotechnology Co. Ltd. in Beijing, China. The rats were maintained in standard cages at a regulated temperature of 24°C and 65% humidity, with unrestricted access to standard rodent chow and water. A 3‐day acclimatization period was provided for the rats. All procedures involving the rats were conducted in strict adherence to the Guidelines for the Care and Use of Laboratory Animals established by the National Academy of Sciences to ensure minimal discomfort and pain to the animals. The experimental protocols got permission from the Animal Care and Use Committee of the Institute of Material Medica Integration and Transformation for Brain Disorders (No. IBD2021013).

### 2.4 Identification and Comparative Analysis of the Key Chemical Components of FA, WB‐FA, TBB‐FA Using UPLC‐Q‐Orbitrap HRMS

10 mL of the FA, WB‐FA, and TBB‐FA solutions, prepared as Section 2.2, were each combined with an additional 10 mL of water and thoroughly mixed. Subsequently, 100 μL of this suspension was taken and mixed with 300 μL of methanol. This mixture was then subjected to a vortex for a duration of 2 min. Following this, the solution was centrifuged at a temperature of 4°C and a speed of 497.67 g for a period of 15 min. The supernatant was then carefully transferred into a pre‐lined tube for further analysis using UPLC‐Q‐Orbitrap HRMS to detect chemical components.

The separation through chromatography was conducted using a Thermo Scientific AccucoreTM C18 column (100 × 3 mm, 2.6 μm) with a mobile phase consisting of water‐0.1% formic acid solution (eluent A) and acetonitrile (eluent B) at a flow rate of 0.3 mL·min^−1^. The gradient program was established as follows: 0–15 min, 5%–30% B; 15–35 min, 30%–70% B; 35–50 min, 70%–95% B; 50–60 min, 95%–95%. The column temperature was maintained at 30°C, and the injection volume was 5 μL.

Electrospray ionization (ESI) was utilized for acquiring the MS data in both positive and negative ion scan modes. The spray voltage was set at +3.5/−3.0 kV, with sheath gas at 40 arb, auxiliary gas at 10 arb, heater temperature at 350°C, and capillary temperature at 320°C. The scanning mode encompassed Full MS/dd‐MS^2^, with the full MS scan ranging from *m*/*z* 100 to 1000 and a resolution of 35,000. For MS/MS scanning mode, a data‐dependent MS^2^ scan was employed with a resolution of 17,500, and high collision‐induced dissociation (HCD) was configured in stepped mode (20, 40, 60 eV).

The raw data in the form of ×.raw files from UPLC‐Q‐Orbitrap HRMS (Q‐Exactive, Thermo Fisher, USA) analysis were captured using Xcalibur (Thermo Fisher, USA), containing details such as retention time, exact mass (*m*/*z*), MS or MS/MS intensity of peak, and fragment ions. Subsequent data processing steps including peak detection, deconvolution, peak alignment, gap filling, and normalization were carried out using Compound Discoverer 3.0 (Thermo Fisher).

### 2.5 Pharmacodynamic Evaluation

Forty rats were randomly assigned to five distinct groups, each comprising eight animals. These groups included a control group, a model group, a group treated with FA extract, a group treated with FA extract combined with WB (WB‐FA), and a group treated with FA extract combined with TBB (TBB‐FA). The model group was subjected to functional dyspepsia by intraperitoneal injection of reserpine solution at a dose of 5 mg/kg over a 20‐day period. Physiological disparities between the model and control groups were monitored at regular intervals during the modeling process. Following successful model preparation, intragastric administration of the respective drugs was initiated with dose gradients set for each treatment group over an 8‐day period. The dosage administered to the groups receiving FA, WB‐FA, and TBB‐FA was 3.25 g/kg. The control group and model group were given an equivalent amount of 0.5% CMCNa solution.

Serum levels of gastrointestinal hormones, including vasoactive intestinal peptide (VIP), substance‐P (SP), and motilin (MTL), were assessed by ELISA kits (Elabscience Biotechnology Co. Ltd., Wuhan, China) using 4 mL of blood collected from the abdominal aorta, which was then centrifuged at 124.61 g for 10 min at 4°C. The resulting supernatant was stored at −80°C for subsequent analysis following the manufacturer's protocols.

### 2.6 Cecum Microbiota Analysis and Assessment of SCFA Levels

#### 2.6.1 Cecal Microbiota Analysis

Initially, the DNA was extracted from the sample, followed by the design and synthesis of the primer adapter, PCR amplification, product purification, quantification, and homogenization of the PCR products. Subsequently, a library was constructed and quantified using Qubit and Q‐PCR methods. Upon library qualification, PE250 on‐machine sequencing was performed using the NovaSeq6000. The Alpha diversity index was then assessed at the OTU level, encompassing metrics such as Shannon, Simpson, and Coverage indices. To delve deeper into species differences and similarities among groups, Beta diversity analysis, UPGMA cluster analysis, and dimensionality reduction techniques including principal component analysis (PCA), principal coordinate analysis (PCoA), and non‐metric multidimensional scaling (NMDS) were conducted for each sample group. Community structure analysis was then carried out by statistically analyzing the proportion of sequence numbers to total sequences based on OTU absolute abundance and annotation information at phylum and genus levels. Differences in species composition at these levels were determined for each group, with inter‐group comparisons at the phylum and genus levels conducted using Student's *t*‐test method. By applying an LAD value of & gt; 4, distinct bacterial groups at the phylum to genus levels were identified through analysis. Finally, Spearman algorithm was employed to conduct a correlation analysis of environmental factors.

#### 2.6.2 Cecal SCFAs Assessment by UPLC‐QQQ/MS/MS

##### 2.6.2.1 (1) Extraction and Derivatization of SCFAs From Feces

The fecal sample solution was formulated by amalgamating 40 mg of fecal material (wet weight) with 200 μL of 60% acetonitrile. The mixture was agitated for 3 min to ensure homogeneity, followed by centrifugation at 435.46 g and 4°C for 10 min to collect the supernatant. Subsequently, 100 μL of the fecal sample solution was mixed with 20 μL of 200 mmol·L^−1^ 3‐NPH·HCl solution, and then 20 μL of 120 mmol·L^−1^ EDC·HCL‐6% pyridine solution was added. The resulting solution was allowed to incubate at 30°C for 30 min, followed by rapid cooling for 1 min and the addition of 10% acetonitrile to a final volume of 2 mL to obtain the fecal sample derivative solution. Prior to injection analysis, 50 μL of the internal standard (2‐methylvaleric acid) was amalgamated with 950 μL of the derivatization solution and centrifuged at 497.67 g for 10 min at 4°C. Subsequently, 500 μL of the supernatant was extracted and placed into a sample bottle for measurement.

##### 2.6.2.2 (2) Chromatographic and Mass Spectrometry Condition

In this study, the UPLC‐QQQ/MS/MS (TSQ‐fortis, Thermo Fisher) Chromato‐mass spectrometry system was utilized for detection and analysis purposes. Chromatographic separation was conducted on a Thermo Scientific AccucoreTM C_18_ column (100 × 3 mm, 2.6 μm) using a mobile phase consisting of water with 0.1% formic acid (eluent A) and acetonitrile (eluent B) at a flow rate of 0.3 mL·min^−1^. The gradient program was structured as follows: 0–2 min, 15% B; 2–10 min, 15%–30% B; 10–18 min, 30%–40%; 18–23 min, 40%–55%; 23–25 min, 55%–95%; 25–27 min, 95%. The column temperature was maintained at 30°C, and the injection volume was 5 μL. The MS data acquisition was carried out using electrospray ionization (ESI) in the positive ion scan modes with specific parameters such as a spray voltage of +3.5/−3.5 kV, sheath gas at 35 arb, auxiliary gas at 15 arb, heater temperature at 350°C, and capillary temperature at 350°C. The scanning mode employed was SRM, and the compound optimization parameters are detailed in Table 1.

**TABLE 1:** MS Parameters of components to be detected.

Compound	Polarity	Precursor (*m*/*z*)	Product (*m*/*z*)	Collision energy (*V*)
Formic acid	Negative	180	137	20
Acetic acid	Negative	194	137	20
	Negative	194	180	20
Propionic acid	Negative	208	137	20
	Negative	208	190	20
Isobutyric acid	Negative	222	137	20
Butyric acid	Negative	222	137	20
	Negative	222	204	20
Lactic acid	Negative	224	137	17
	Negative	224	152	14
Trimethylacetate	Negative	236	137	20
Valeric acid	Negative	236	137	20
Isovaleric acid	Negative	236	137	20
	Negative	236	218	20
4‐Methylvalerate	Negative	250	137	20
Hexanoate	Negative	250	137	20
2‐Methylvalerate (IS)	Negative	250	137	20
	Negative	250	232	20
2‐Methylhexanoate	Negative	264	137	20
Heptanoate	Negative	264	137	20
	Negative	264	246	20
Pyruvate	Negative	357	137	20
Methylmalonate	Negative	387	150	21
	Negative	387	177	16
Succinic acid	Negative	387	190	20
	Negative	387	233	16

##### 2.6.2.3 (3) Methodological Validation of Quantitative Analysis

The standard solution for each short‐chain fatty acid was prepared at a concentration of 1 mol/L, with 50 μL of each solution combined in a single EP tube. Subsequently, 200 μL of a 60% acetonitrile solution was added to create a mixed solution with a maximum concentration of 500 μM/L, which was then diluted incrementally to 0.5 μM/L. Following the optimal derivatization reaction conditions, a 5 μL aliquot of the mixed standard solution was precisely measured and mixed with an internal standard. This mixture was then injected into the UPLC‐QQQ‐MS/MS system for detection in SRM mode, and the resulting peak area was calculated. By plotting the ratio of each short‐chain fatty acid concentration to the internal standard concentration on the horizontal axis (*X*) against the ratio of peak areas on the vertical axis (*Y*), a standard curve was generated along with the regression equation.

To assess precision, a 500 μM/L mixed standard solution was injected three times consecutively to determine the peak area and calculate the intraday precision as the relative standard deviation (RSD). Similarly, the mixed solution was injected twice daily for three consecutive days to determine the daily precision, also expressed as RSD. The stability of the fecal sample solution prepared under optimal conditions was evaluated by measuring the peak area at various time points (0, 2, 8, 10, 18, and 24 h) and expressing the results as RSD. Reproducibility was assessed by preparing six parallel fecal sample solutions, measuring their peak areas, and calculating the RSD. Finally, 20 μL of fecal solution was taken, and varying amounts (50%, 100%, and 150%) of control products were added. The resulting peak areas were determined, and the recovery rate was calculated and expressed as RSD.

### 2.7 Untargeted Metabolomics Analysis

#### 2.7.1 Preparation of Intestinal Mucosa Samples

Following the weighing and recording of intestinal mucosa samples from liquid nitrogen, the samples were immediately ground with liquid nitrogen. Then, 800 μL of 80% chromatographic grade methanol was added to a 1.5 mL Eppendorf tube. The mixture was vortexed and agitated at low temperature, then incubated at −80°C for 4 h, centrifuged at 435.46 g, 4°C, for 10 min. The resulting supernatant was taken out and transferred to a fresh 1.5 mL microcentrifuge tube. Next, 400 μL of precooled 80% methanol was added to the remaining precipitate, which was then vortexed, mixed, incubated at −80°C for 30 min, and centrifuged at 4°C at 435.46 g for 10 min. The supernatant obtained from two consecutive transfers was subjected to another round of centrifugation at 435.46 g, 4°C, for 10 min. This supernatant was then moved to a new 1.5 mL microcentrifuge tube and freeze‐dried at low temperature. Subsequently, a 30 μL precooled 500 ng/mL solution was added to the desiccated metabolite sample as the internal standard. The mixture was pipetted and swirled at low temperature for 1 min, followed by centrifugation at 513.22 g for 5 min at 4°C. Finally, 20 μL of the resulting supernatant was drawn into the liner tube for mass spectrometry analysis.

#### 2.7.2 Preparation of Serum Samples

400 μL of 80% methanol at a low temperature was combined with 80 μL of serum, vortexed for 30 s, incubated at 4°C for 30 min, and then centrifuged at 16,500 rpm for 5 min at 4°C. The resulting supernatant was transferred to a new centrifuge tube, and 30 μL of a pre‐cooled 500 ng/mL dichlorophenylalanine solution was added as an internal standard. The mixture was vortexed for 1 min at a low temperature, followed by hand shaking on ice for 3 min. Subsequently, the solution was centrifuged at 16,500 rpm for 5 min at 4°C, and 20 μL of the supernatant was drawn into a liner tube, which was then positioned vertically in a brown chromatographic flask for mass spectrometry analysis.

#### 2.7.3 Chromatographic and Mass Spectrometry Condition

Chromatographic and mass spectrometry conditions are the same as 2.4.

#### 2.7.4 Metabolomics Data Processing

The raw mass spectrometry (MS) data were imported into MZmine 2.49 software to obtain a dataset of all the ions and their intensities for all samples (Schmid et al. 2023). This dataset was subsequently utilized for various multivariate data analysis techniques, such as principal component discriminant analysis (PCA‐DA), partial least squares discriminant analysis (PLS‐DA), orthogonal partial least squares analysis (OPLS‐DA), and unsupervised cluster analysis, to detect and characterize distinct metabolites and potential biomarkers, as well as to conduct enrichment analysis of their metabolic pathways (Brigante et al. 2021). Biomarker information was obtained by searching databases such as KEGG, HMDB, METLIN, LIPIDMAPS, etc. The identified metabolites were further analyzed using metabolic analyzer 3.0 for enrichment purposes.

### 2.8 Statistical Analysis

In the statistical analysis section, the findings were reported in the form of mean values with standard deviations. Data analysis was conducted using GraphPad Prism 8.0.1 statistical software. Student's *t*‐test was employed for comparing data between two groups, while the one‐way ANOVA method was utilized for comparing data across multiple groups. Additionally, GraphPad Prism 8.0.1 in conjunction with Origin nine was used for further analysis.

## 3 Results

### 3.1 Detection and Comparative Analysis of Distinctive Chemical Components of FA and Two Distinct Formulations Using UPLC‐Q‐Orbitrap HRMS

**FIGURE 1:**
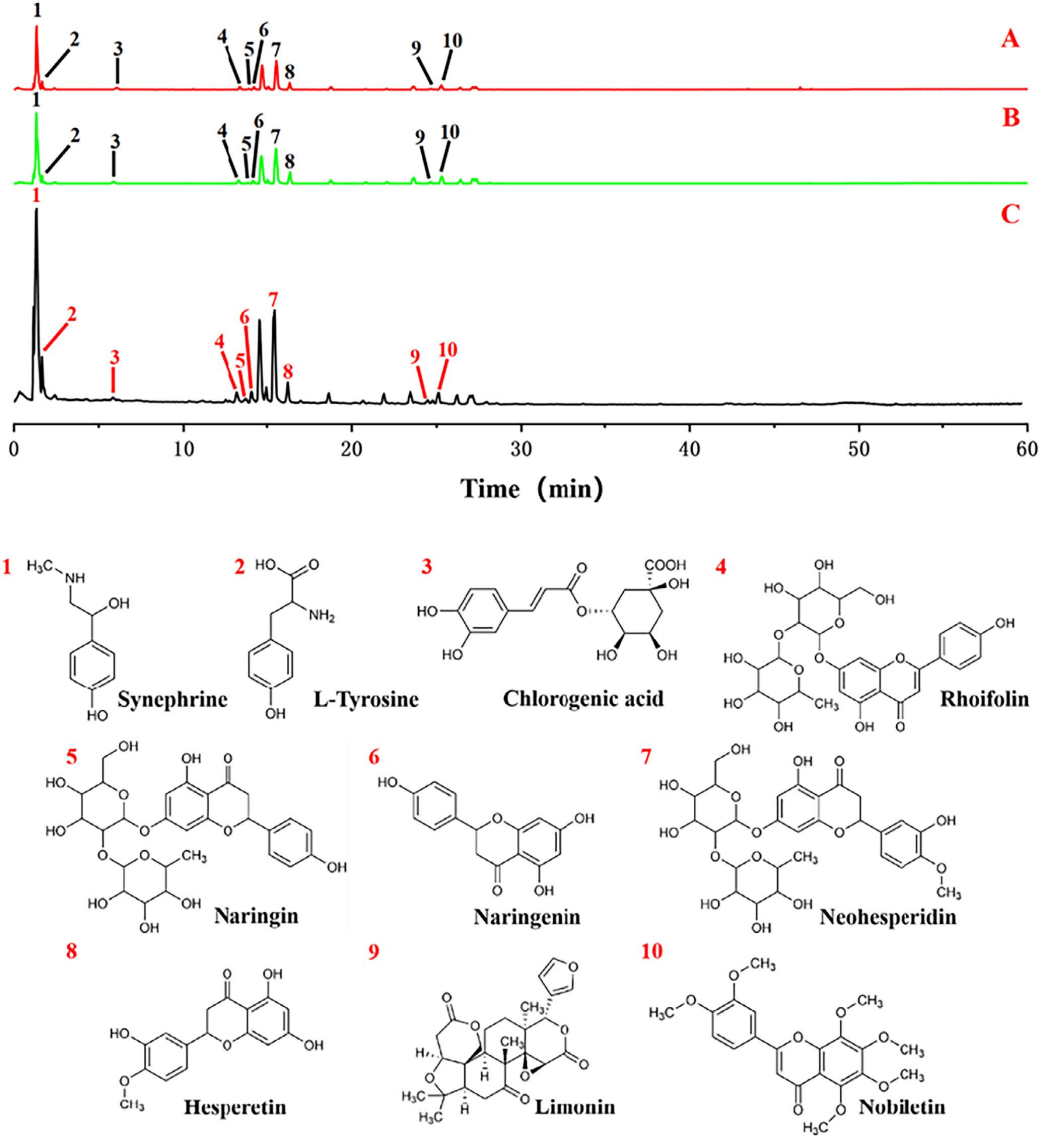
Chemical components of fructus aurantii and its two products analyzed by UPLC‐Q‐Orbitrap HRMS (TIC diagram of positive ions). (A) FA. (B) WB‐FA. (C) TBB‐FA.

**FIGURE 2:**
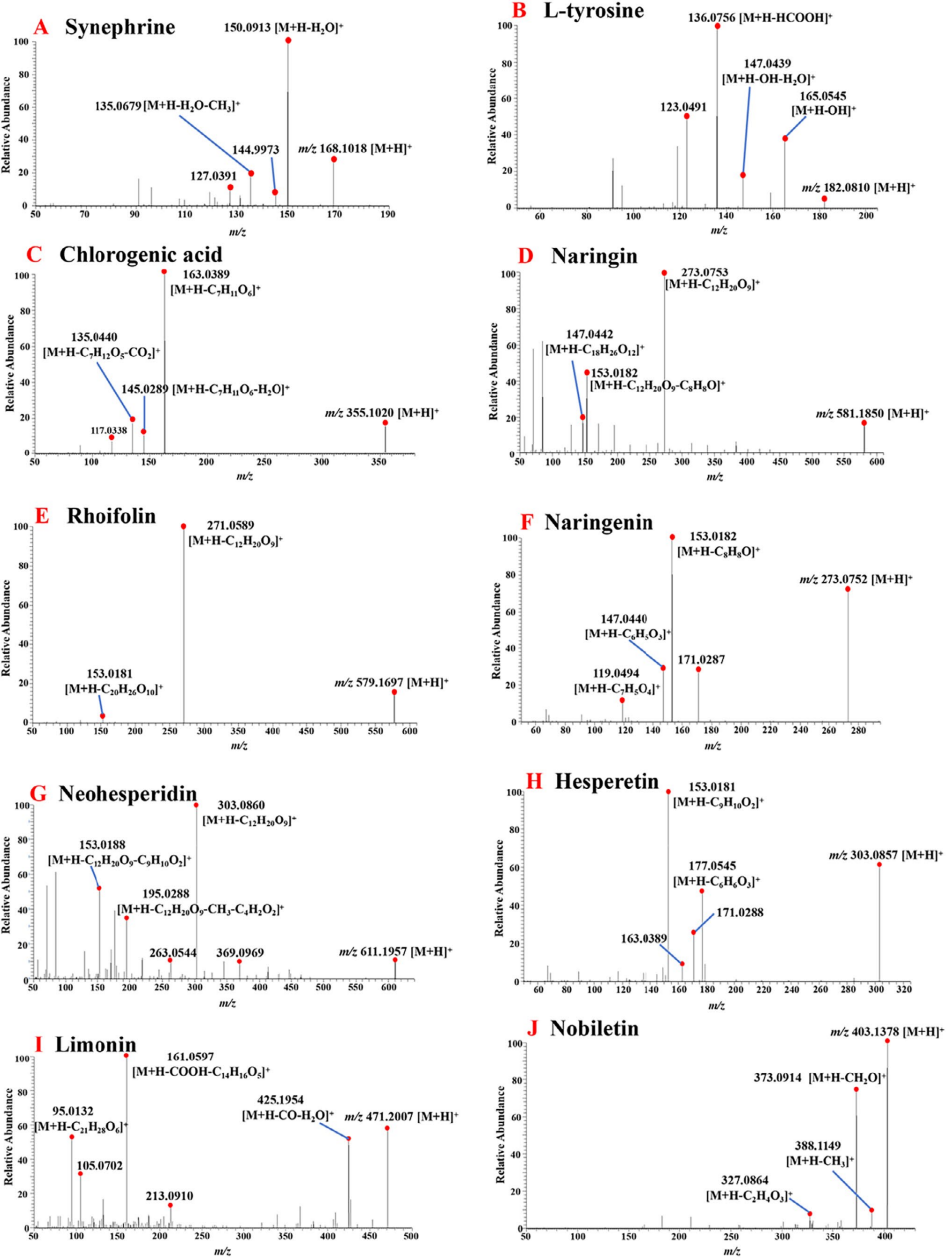
10 secondary fragments of Q‐Markers associated with FD in FA.

The total ion chromatograms (TIC) of FA and two products obtained by UPLC‐Q‐Orbitrap HRMS are shown in Figure 1. The 10 index compounds, which were used as quality markers for FA, and their relative peak area values are listed in Table 2. The extraction ion flows and primary fragment fragmentation patterns of these compounds are depicted in Figure 2. Notably, discernible variations in the relative abundance of certain ion peaks were observed based on the TIC profiles.

**TABLE 2:** Identification and comparison of chemical constituents of FA, WB‐FA, and TBB‐FA by UPLC‐Q‐Orbitrap HRM.

No.	Compound	RT/min	Ion mode	Molecular weight	Chemical formula	*δ*/*p*pm	Major fragment ion	Relative peak area		
								FA	WB‐FA	TBB‐FA
1	Synephrine	1.35	(M + H)+	168.1018	C_9_H_13_NO_2_	0.59	150.0913, 144.9973, 135.0679, 127.0391	2.95 × 10^8^	3.65 × 10^8^	3.48 × 10^8^
2	L‐tyrosine	1.40	(M + H)+	182.0810	C_9_H_11_NO_3_	0.55	165.0545, 147.0439, 136.0756, 123.0491	1.43 × 10^8^	1.91 × 10^8^	2.12 × 10^8^
3	Chlorogenic acid	5.66	(M + H)+	355.1020	C_16_H_18_O_9_	−1.13	163.0389, 145.0284,135.0440, 117.0338	1.28 × 10^8^	7.16 × 10^7^	2.10 × 10^8^
4	Naringin	14.19	(M + H)+	581.1850	C_27_H_32_O_14_	−2.58	273.0753, 153.0182, 147.0442	6.71 × 10^9^	3.93 × 10^8^	8.09 × 10^9^
5	Rhoifolin	14.61	(M + H)+	579.1697	C_27_H_30_O_14_	1.07	271.0598, 153.0181, 119.0493	3.02 × 10^8^	1.54 × 10^8^	4.75 × 10^8^
6	Naringenin	14.65	(M + H)+	273.0752	C_15_H_12_O_5_	−2.20	153.0182, 171.0287, 147.0440	7.44 × 10^9^	8.44 × 10^9^	9.31 × 10^9^
7	Neohesperidin	15.51	(M + H)+	611.1957	C_28_H_34_O_15_	−2.13	303.0860, 195.0288, 153.0188, 263.0544, 369.0969	5.33 × 10^9^	9.29 × 10^9^	6.59 × 10^9^
8	Hesperetin	15.54	(M + H)+	303.0857	C_16_H_14_O_6_	−1.98	153.0181, 177.0545, 171.0288, 163.0389	8.73 × 10^9^	6.50 × 10^9^	1.24 × 10^10^
9	Limonin	24.64	(M + H)+	471.2007	C_26_H_30_O_8_	−1.27	425.1954, 161.0597, 95.0132, 105.0702, 213.0910	8.38 × 10^8^	1.12 × 10^10^	1.35 × 10^9^
10	Nobiletin	25.28	(M + H)+	403.1378	C_21_H_22_O_8_	−2.23	373.0914, 388.1149, 327.0864	1.29 × 10^9^	1.47 × 10^9^	2.45 × 10^9^

### 3.2 Pharmacodynamic Assessment

#### 3.2.1 Observations of Overall Behavior and Changes in Body Weight

**FIGURE 3:**
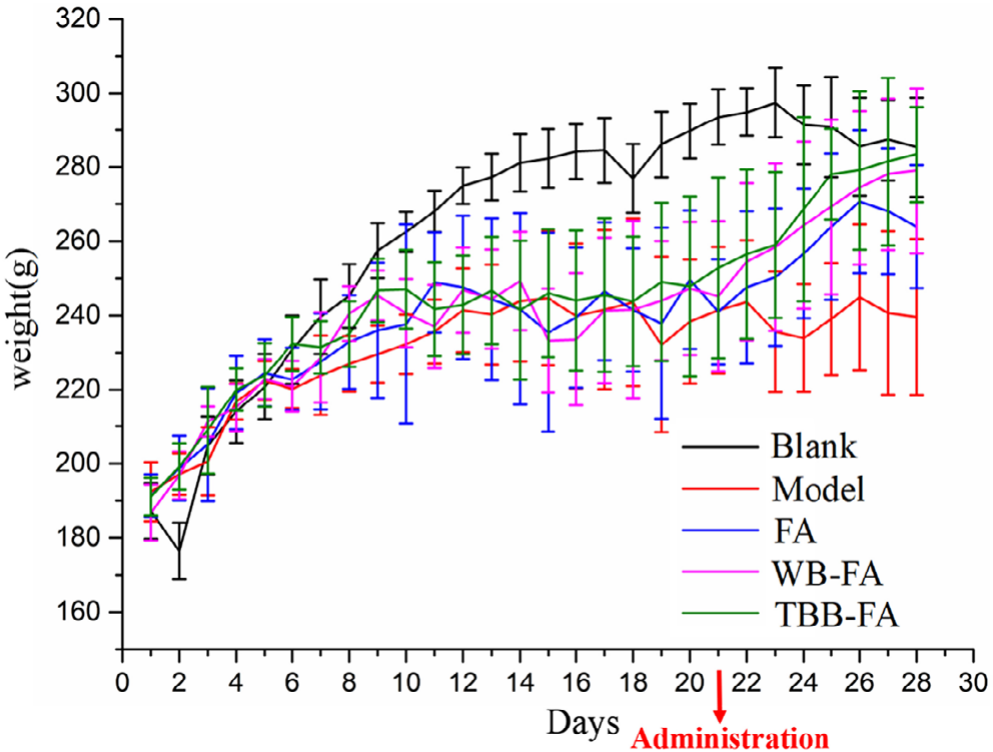
Body weight change curve of each group before and after administration.

From the fifth day of the modeling, a significant reduction in weight gain was observed in rats compared to the control group. Additionally, the rats exhibited symptoms including yellowing fur, soft feces, diminished mental state, and heightened irritability, thereby confirming the successful establishment of the model. The modeling was carried out for 20 days, after which the drug was administered over a period of 8 days on day 21. Subsequent to the intervention with FA, WB‐FA, and TBB‐FA, improvements were observed in the rats, including the restoration of fur luster, dry feces, and a more placid temperament. Notably, the weight gain in the group treated with TBB‐FA showed a slightly superior outcome compared to other treatment groups, as depicted in Figure 3.

#### 3.2.2 Determination of Serum Levels of Gastrointestinal Hormones

**FIGURE 4:**
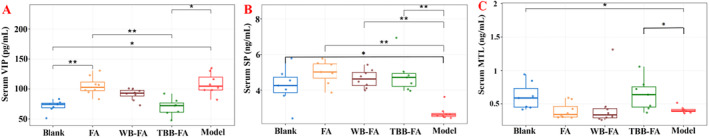
Changes of serum gastrointestinal hormone levels in rats. (A) VIP, (B) SP, (C) MTL. *p < 0.05, with significant difference, **p < 0.01, with extremely significant difference.

The serum concentrations of gastrointestinal hormones in rats were evaluated subsequent to the administration of FA, WB‐FA, and TBB‐FA. Figure 4 shows that the VIP, SP, and MTL levels in the model group were significantly different from those in the control group (*p* < 0.05). Upon treatment with TBB‐FA, a significant increase in serum SP and MTL levels was observed in comparison to the model group (*p* < 0.05), while VIP levels were notably suppressed. The therapeutic efficacy was marginally superior or comparable to that of the WB‐FA group.

#### 3.2.3 16S rRNA Sequencing Assay

**FIGURE 5:**
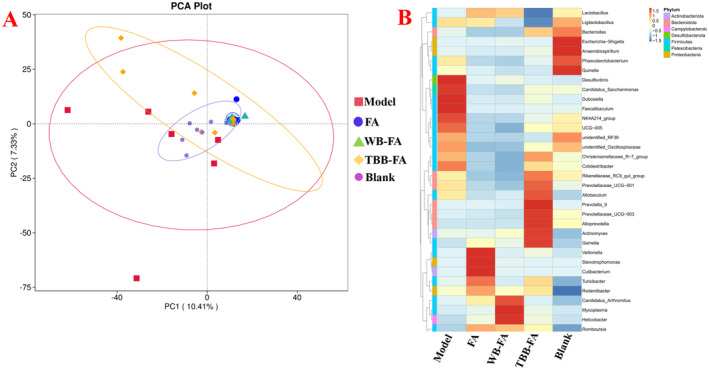
16S rRNA sequencing analysis. (A) PCA, (B) Heat map of intestinal flora.

The 16S rRNA sequencing assay was employed to explore the potential relationship between the effectiveness of TBB‐FA in mitigating FD and modifications in intestinal flora. The results of the principal component analysis (PCA) are depicted in Figure 5A. The control group is situated at the center of the coordinate axis, whereas the model group exhibits a collective shift towards the lower left. The observed alterations in intestinal flora following FD modeling are minimal, potentially aligning with the pathogenesis of FD. This suggests that significant changes in intestinal flora may not occur during the onset of FD. In addition, it also suggests that there are significant individual differences in the microbial community. The FA group and the WB‐FA group are located in the upper right of the PCA plot, and the difference between these two groups and the blank group is larger than the difference between the model group and the blank group. After administration of TBB‐FA, although the flora of each rat was significantly different (orange spots were widely dispersed). However, on the whole, the coincidence between TBB‐FA group and blank group was still higher. The results showed that the expression of some bacteria had significant reversal. As shown in Figure 5B, the expression of some bacteria genera showed a reversal, such as Bacteroides, *Escherichia coli* , etc. Meanwhile, 16S rRNA sequencing results (Figure 5B) showed that compared with the blank group, the overall gut flora abundance of the model group had little change, but the expression of individual bacteria genera had substantial changes compared with the blank group, such as acteroides, escherichia‐Shigella, anaerobiospirilum, and phascolarctobacterium expression abundance decreased 4.18, 45.25, 16.58 and 1.71 times compared with blank group, respectively. In contrast, the expression abundance of Desulfovibrio, candidatus_saccharimonas, and dubosiella was significantly increased in the model group, with the expression of Desulfovibrio increasing nearly seven times. Bacteroides emerged as a key player in the recovery and regulation of FA stir‐baking with TBB compared to the model group. Additionally, the abundance expressions of colidextribacter ( *Escherichia coli* ), allobaculum, and alloprevotella significantly increased and surpassed those of the blank group (*p* < 0.01).

#### 3.2.4 Analysis of SCFAs Level by UPLC‐QQQ/MS/MS

##### 3.2.4.1 (1) Spectrogram

**FIGURE 6:**
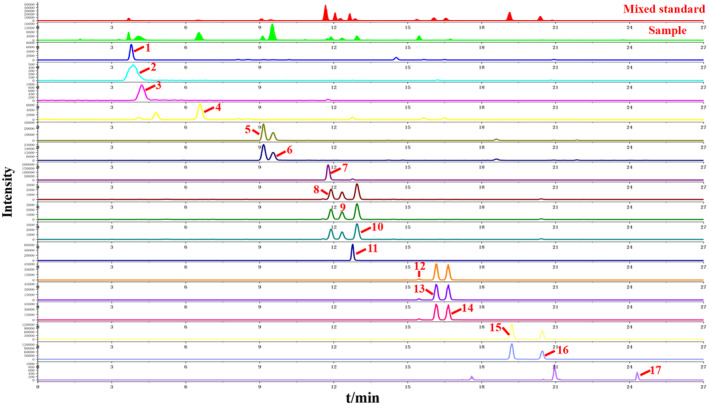
The TIC chromatogram of the mixed standard, sample, and EIC chromatogram of each short‐chain fatty acid. (1) Lactic acid, (2) Formic acid, (3) Acetic acid, (4) Propionic acid, (5) Isobutyric acid, (6) Butyric acid, (7) Succunic acid, (8) Isovaleric acid, (9) Trimethylacetate, (10) Valeric acid, (11) Methylmalonate, (12) 2‐Methylvalerate (IS), (13) 4‐Methylvalerate, (14) Hexanoate, (15) 2‐Methylhexanoate, (16) Heptanoate, (17) Pyruvate.

The spectrograms were obtained by UPLC‐QQQ/MS/MS analysis of SCFA levels. Figure 6 displays the total ion current (TIC) chromatogram of the combined standard and sample in negative ion mode, as well as the extracted ion current (EIC) chromatogram for each individual SCFA.

##### 3.2.4.2 (2) Validation of Quantitative Methods

The peak area of each control exhibited a strong linear correlation with concentration, with an *R*‐squared value exceeding 0.9800, as detailed in Table 3. The intraday and interday precision, with relative standard deviations (RSD) below 4.8%, demonstrated the high precision of the instrument. Results from reproducibility experiments indicated that the RSD of peak area was below 5.3%, and within a 24‐h period, the RSD of peak area remained under 6%, affirming the method's reproducibility and stability. Furthermore, the recovery rate falling within the range of 80%–120% confirmed the accuracy of the method.

**TABLE 3:** Linear relationship of short‐chain fatty acids to be measured.

No.	Analyte	Calibration curve	*R* ^2^	Linear range/μmol L^−1^
1	Lactic acid	*Y* = 1.12^−3^ *X* + 1.99131	0.9970	0.5–500
2	Formic acid	*Y* = 2.42316^−4^ *X* + 0.06733	0.9911	0.5–500
3	Acetic acid	*Y* = 2.80581^−4^ *X* + 0.01423	0.9811	0.5–500
4	Propionic acid	*Y* = 1.28 × 10^−3^ *X* + 0.01926	0.9932	0.5–500
5	Isobutyric acid	*Y* = 3.55^−3^ *X* + 0.0126	0.9997	0.5–500
6	Butyric acid	*Y* = 1.15^−3^ *X* + 0.00142	0.9998	0.5–500
7	Succinic acid	*Y* = 2.864^−2^ *X* + 0.51181	0.9966	0.5–500
8	Hexanoate	*Y* = 4.62^−3^ *X* − 6.69683E^−4^	0.9999	0.5–500
9	2‐Methylhexanoate	*Y* = 1.852^−2^ *X* − 0.04916	0.9992	0.5–500
10	Heptanoate	*Y* = 1.853^−2^ *X* − 0.05048	0.9992	0.5–500
11	Trimethylacetate	*Y* = 8.08 × 10^−3^ *X* + 0.01202	0.9999	0.5–500
12	Isovaleric acid	*Y* = 3.14 × 10^−3^ *X* + 0.00781	0.9999	0.5–500
13	Methylmalonate	*Y* = 5.97 × 10^−3^ *X* + 0.09451	0.9945	0.5–500
14	Valeric acid	*Y* = 3.7 × 10^−3^ *X* + 0.03174	0.9974	0.5–500
15	4‐Methylvalerate	*Y* = 4.97 × 10^−3^ *X* − 0.00775	0.9998	0.5–500
16	Pyruvate	*Y* = 8.13624E^−4^ *X* + 0.00706	0.9993	0.5–500
17	2‐Methylvalerate (IS)	*Y* = 9.14423 × 10^−19^ *X* + 1	—	0.5–500

##### 3.2.4.3 (3) Determination of Content

**FIGURE 7:**
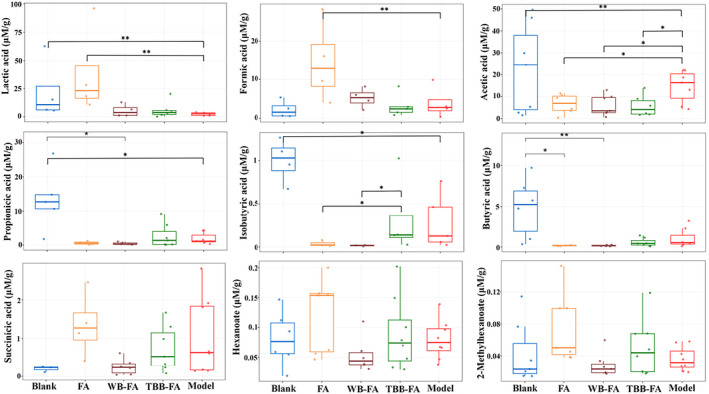
Expression levels of 9 SCFAs. *p < 0.05, with significant difference, **p < 0.01, with extremely significant difference.

The SCFA content in the fecal samples of rats in all experimental groups was analyzed. It was observed that the levels of nine SCFAs differed significantly among the groups, with some exhibiting notable variances (*p* < 0.05), as depicted in Figure 7. In comparison to the control group, the concentrations of lactate, acetate, propionate, and isobutyrate were markedly reduced in the model group (*p* < 0.05). After treatment, the administration of FA resulted in a significant reduction in lactate and formate levels compared to the model group (*p* < 0.01). Furthermore, the propionate, butyrate, and isobutyrate contents in rat feces were significantly higher after TBB‐FA group consumption (7.15, 33.2, and 3.77 times higher than those in WB‐FA group). Previous research has indicated that propionate and butyrate are the primary metabolic byproducts of Bacteroides, *Escherichia coli* , and Prevotella (Fan and Pedersen 2020). Importantly, the results of 16S rRNA sequencing also indicated a significant elevation in the abundance of these three strains when FA was stir‐fried with TBB. These findings were consistent across the analyses.

### 3.3 Non‐Targeted Metabolomics Analysis of Serum and Intestinal Mucosa

**FIGURE 8:**
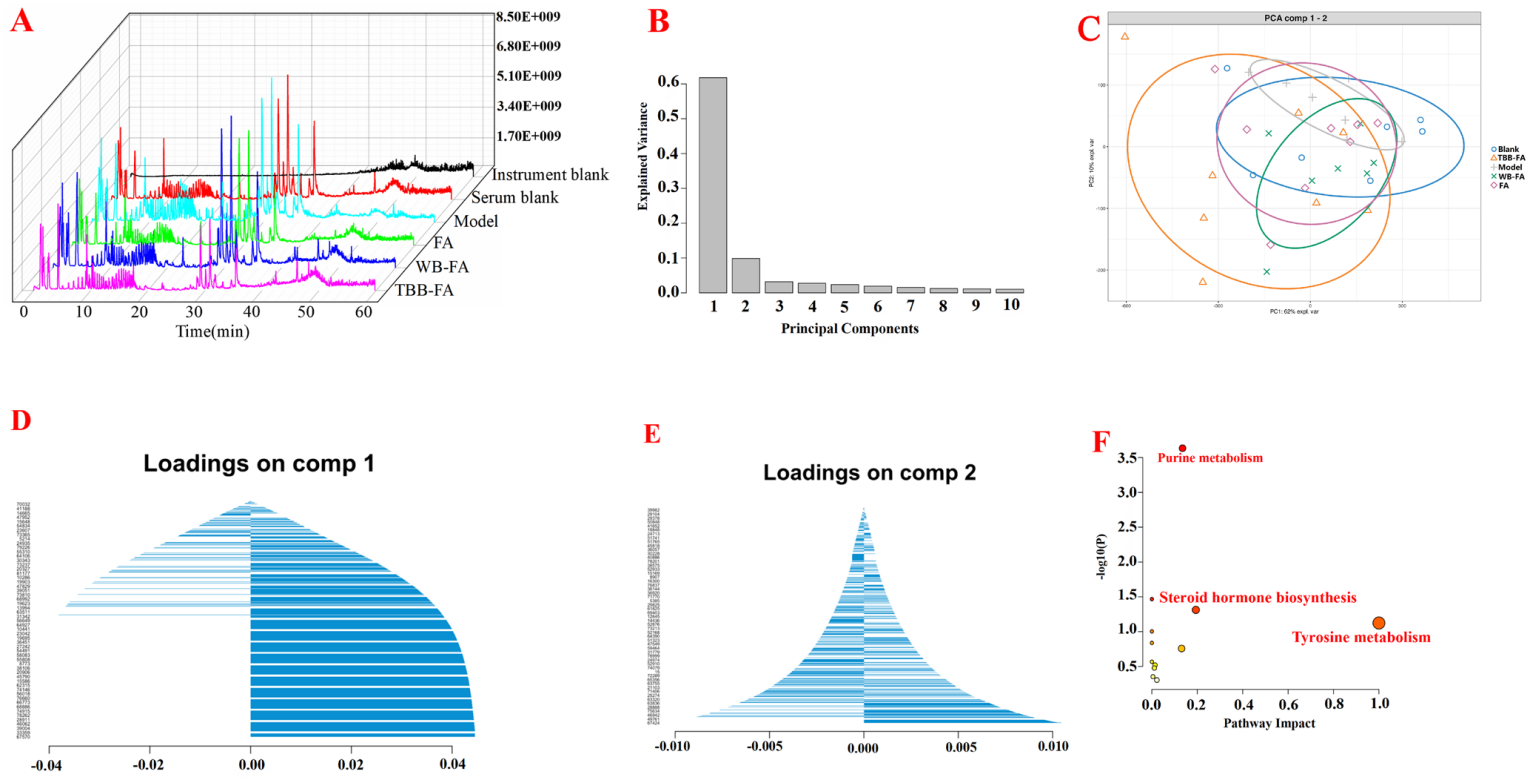
Metabolomics analysis of blood serum from FD rats given different processed FA. (A) Total ion flow diagram; (B) Principal component quantity distribution map; (C) PCA diagram; (D) The first component is specific metabolite map; (E) The second component is specific metabolite map; (F) Enrichment analysis diagram of metabolic pathway.

In the non‐targeted metabolomics analysis of serum, the results indicated a significant abundance of metabolites in each group, as depicted in Figure 8A. The findings from the serum PCA demonstrated a classification trend analogous to that of the intestinal flora, with significant differences observed between groups, as illustrated in Figure 8C. It is worth noting that the TBB‐FA group exhibited a distinct deviation and higher concentration relative to the other drug administration groups. A total of 23 potential biomarkers associated with FD were identified, including Adenine, L‐Dopa, and Cortisol. The enrichment analysis of the functional pathways associated with these biomarkers revealed that Adenine is linked to purine metabolism, L‐Dopa and 4‐Hydroxyphenylpyruvic acid are related to tyrosine metabolism, and Cortisol is connected to the steroid hormone biosynthesis pathway.

**FIGURE 9:**
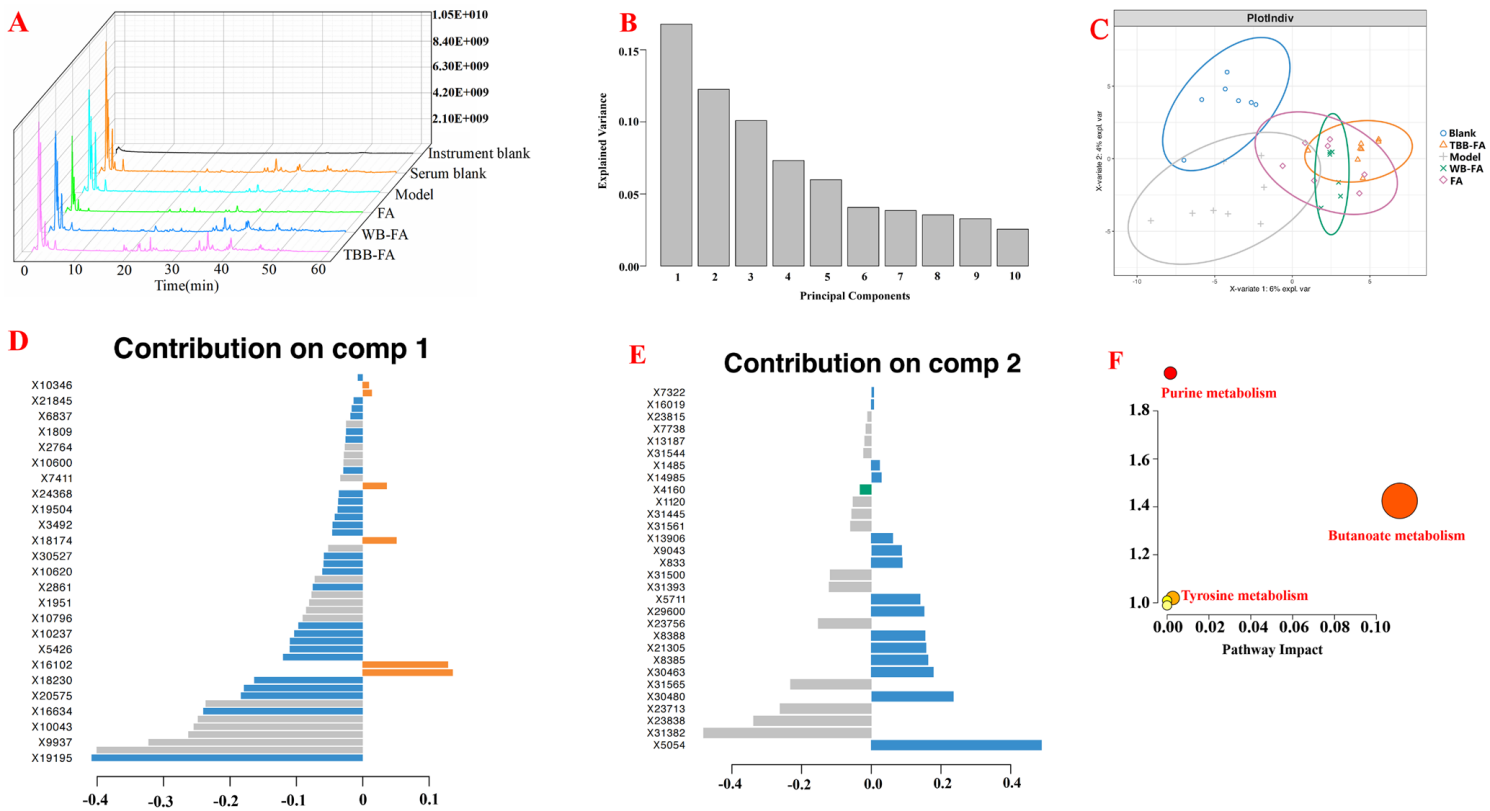
Metabolomics analysis of intestinal mucosa from FD rats given different processed FA. (A) Total ion flow diagram; (B) Principal component quantity distribution map; (C) PCA diagram; (D) The first component is specific metabolite map; (E) The second component is specific metabolite map; (F) Enrichment analysis diagram of metabolic pathway.

Similarly, the analysis of intestinal mucosal metabolomics revealed a rich abundance of metabolites in each group (Figure 9A). The intestinal mucosal PCA results showed significant deviations in the model group compared to the blank group, with each drug administration group displaying a propensity towards alignment with the blank group. Interestingly, the TBB‐FA group was closer to the blank group, as depicted in Figure 9C. A total of 11 potential biomarkers associated with FD were identified, including 6‐Methylmercaptopurine, Cyclic GMP, Acetylcysteine, Adenosine, Acetoacetic acid, Leukotriene C5, LysoPC, and other phospholipid compounds. The enrichment analysis of their functional pathways disclosed associations such as 6‐Methylmercaptopurine, Adenosine, and Cyclic GMP with purine metabolism. Additionally, Acetoacetic acid was linked to both tyrosine metabolism and butyric acid metabolism.

The findings indicated a consistent involvement of purinergic pathways in both metabolomic analyses. Literature review suggests that the purinergic signaling pathway is implicated in the pathogenesis of gastrointestinal diseases. Modulation of the purinergic signaling pathway has shown potential in enhancing the motility of gastrointestinal smooth muscle cells, promoting gastric emptying. Consequently, it is being investigated as a novel target for the treatment of gastrointestinal motility disorders (Dal Ben et al. 2020).

### 3.4 Correlation Analysis

**FIGURE 10:**
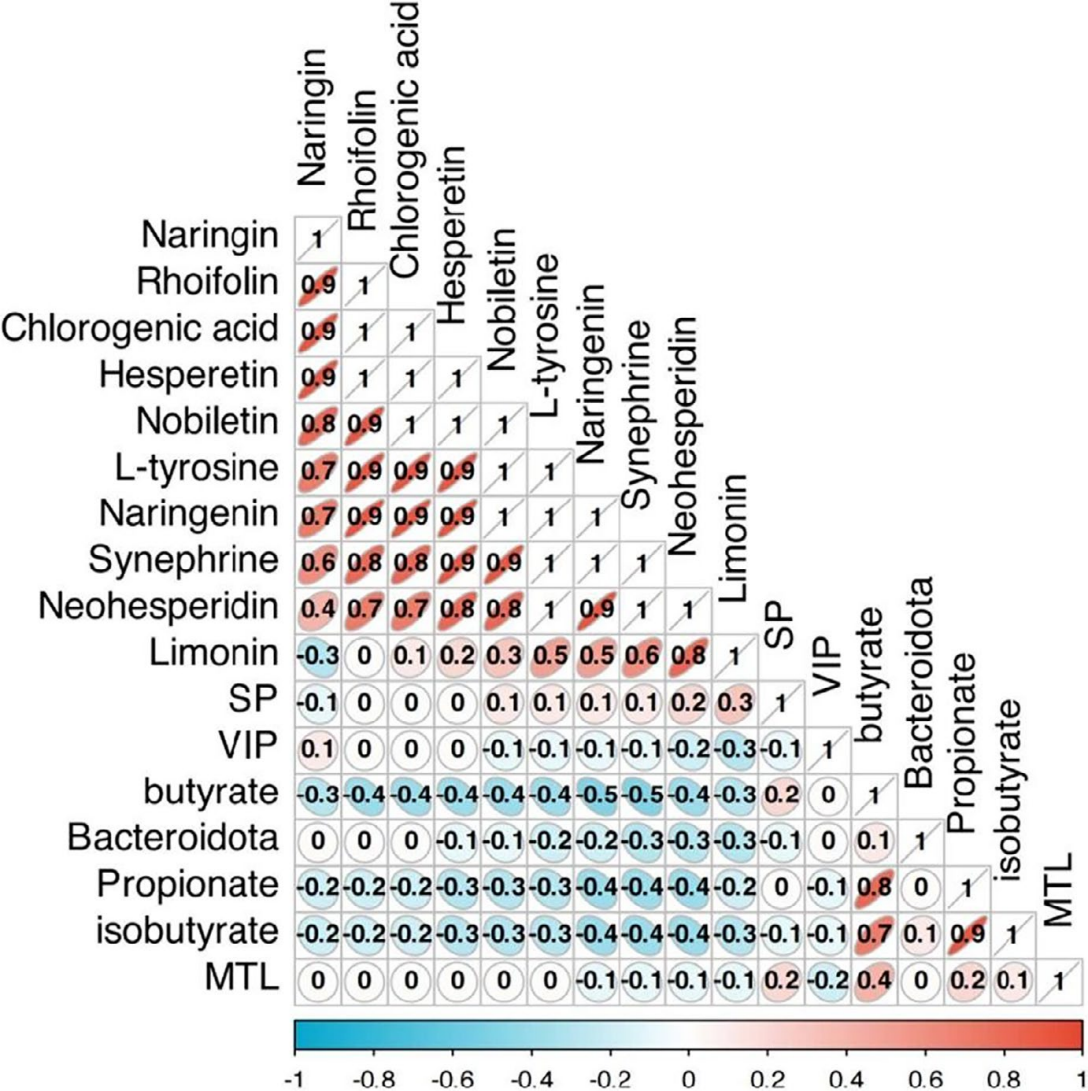
Correlation analysis.

Correlation analysis was performed on the data pertaining to 10 quality markers associated with the treatment of FD using FA, the levels of three gastrointestinal hormones, and the levels of Bacteroides and three SCFAs in fecal samples following the administration of TBB‐FA to FD rats. The analysis revealed a significant positive correlation between Bacteroides and the expression of propionate and isobutyrate (*r* ≥ 0.7), as illustrated in Figure 10. These findings suggest that the combination of fructus aurantii stir‐fried with tartary buckwheat bran may enhance the gut microbiota, elevate the presence of Bacteroides, and stimulate the production of SCFAs like propionate and isobutyrate. Importantly, butyrate has been shown to reduce the production of purine neurotransmitters ATP and NAD^+^, inhibit purinergic signaling pathways, enhance gastrointestinal smooth muscle contractions, and effectively address FD by modulating the glucose metabolism pathway (Yu et al. 2022; Hinrichsen et al. 2021). These outcomes align with the findings presented in Section 3.3.

## 4 Discussion

### 4.1 Investigation Into the Regulatory Mechanism of TBB‐FA on the “Intestinal Flora‐SCFAs‐Metabolic Pathway” in Rats With FD

This study found that after modeling, there was a significant decrease in the abundance of beneficial intestinal bacteria genera such as Lactobacillus, Bacteroides, Escherichia‐Shigella, and Phascolarctobacterium in the intestines of FD rats. These bacteria are known to produce SCFAs such as acetate and butyrate in the gut. Conversely, there was a notable increase in the abundance of harmful intestinal bacteria genera, such as Desulfovibrio, which can produce hydrogen sulfide (H_2_S). Furthermore, upon administering TBB in stir‐fried FA, it was observed that compared to the control group, the expression levels of most beneficial gut bacteria were significantly reduced. Notably, the impact of this correction was significantly better than that of FA and WB‐FA groups. For instance, the expression level of Alloprevotella in the TBB‐FA group was approximately 4 times higher than that in the control group and 16 times higher than that in the WB‐FA group.

Interestingly, these highly abundant intestinal beneficial bacteria share a common trait in that they can produce SCFAs such as propionate and butyrate in the gut after fermentation of dietary fiber and resistant starch. For example, Bacteroides and Colidextribacter are capable of degrading dietary fiber into monosaccharides, primarily pentose and hexose. These bacteria then generate SCFAs through either glycolysis or the pentose phosphate pathways. One of the key pathways in propionic acid synthesis is the succinic acid pathway, with Bacteroides capable of synthesizing propionic acid using pentose or hexose as a substrate (Xu et al. 2020). Additionally, Colidextribacter has the potential to modulate bile acid metabolism and augment butyrate production (Liu et al. 2022). Studies have indicated that butyrate can reduce the production of purine neurotransmitters ATP and NAD^+^, obstruct the purinergic signaling pathway, and stimulate gastrointestinal smooth muscle contraction by modulating the glucose metabolism pathway (Yu et al. 2022; Hinrichsen et al. 2021). Hinrichsen et al. (2021) demonstrated that butyrate can impede the activity of the epigenetic regulatory factor histone deacetylase 8 (HDAC8), thereby down‐regulating the expression of hexokinase 2 (HK2). As HK2 is a crucial metabolic enzyme catalyzing the initial step of the glycolysis pathway, restricting its expression significantly slows down the conversion of glucose to fructose 6‐phosphate, affecting the entire glycolysis process and reducing ATP and NADH production. Given that NADH can swiftly convert to NAD^+^ in the cytoplasm, the amount of NAD^+^ conversion is also limited (Giroud‐Gerbetant et al. 2019). Consequently, it is postulated that an increase in butyrate levels in the intestine inhibits the glycolytic pathway, reduces ATP and NAD^+^ production, and consequently inhibits the purinergic signaling pathway.

Recent studies have validated the association between the purinergic signaling pathway and the pathogenesis of gastrointestinal diseases. Modulating the purinergic signaling pathway can enhance the kinetic energy of gastrointestinal smooth muscle cells, promote gastric emptying, and has been explored as a potential novel target for treating gastrointestinal motility disorders (Dal Ben et al. 2020). Research has also indicated that gastrointestinal dysfunction may be linked to the expression of P2Y1 purine receptors in gastrointestinal smooth muscle (Chang‐Graham et al. 2020; Burnstock 2014; Chang et al. 2023; Zhang et al. 2020). Von Kügelgen (2021) demonstrated that the purine neurotransmitter adenosine triphosphate (ATP) or coenzyme dehydrogenase (NAD^+^) binds to the P2Y1 receptor in gastrointestinal smooth muscle post‐release from intestinal inhibitory neurons, activating phospholipase C‐β through G protein coupling and generating inositol‐1, 4, 5‐triphosphate (IP3). IP3 can boost Ca^2+^ release from the endoplasmic reticulum, activate K^+^ channels, hyperpolarize the membrane potential of gastrointestinal smooth muscle. This process inhibits the kinetic energy of gastrointestinal smooth muscle contraction and alters gastrointestinal motility. Inami, Kiyono, and Kurashima (2018) also confirmed that the purine neurotransmitter ATP or NAD^+^ released from intestinal inhibitory neurons was partially metabolized by ADP‐ribosyl cyclase (CD38) to adenosine diphosphate ribose (ADPR), then to adenosine monophosphate (AMP). AMP is further metabolized to adenosine (ADO) via exonucleoside 5′‐nucleotidase (NT5E)/CD73, which binds to platelet‐derived growth factor (PDGFRα+) and adenosine receptors (AR) in gastrointestinal smooth muscle cells (SMCs) to activate K^+^ channels, leading to cellular hyperpolarization and inhibition of the kinetic energy of gastrointestinal smooth muscle cells. Therefore, the purinergic signaling pathway holds promise as a potential new target for Traditional Chinese Medicine (TCM) treatment of FD.

### 4.2 Research Status of FD‐Related Signaling Pathways Treated by Traditional Chinese Medicine

Currently, TCM treatment of FD involves the modulation of various signal pathways, with a growing emphasis on investigating the associated molecular mechanisms. Notably, the SCF/c‐Kit, 5‐HT, and CRF signaling pathways are commonly utilized in this context, with particular attention given to the regulation of the 5‐HT signaling pathway as a classical mechanism for treating FD (Chang et al. 2023). The 5‐HT signaling system, encompassing the synthesis, release, receptor binding, and reuptake of 5‐HT, plays a crucial role in gastrointestinal function. Dysregulation of this system can lead to motor dysfunction and visceral hypersensitivity, underscoring its significance in functional gastrointestinal disorders (Feng et al. 2023). Studies have shown that interventions such as the Shouxuantongbian capsule and WeitongXiaopi formula can enhance 5‐HT levels in the small intestine by modulating its production, absorption, and degradation, thereby improving gastrointestinal motility and alleviating constipation (Bai et al. 2022; Fan, Zhang, and Chen 2020). Additionally, the activation of purinergic signaling pathways, including excitatory and inhibitory receptors, in response to mechanical stimulation of intestinal pigment cells can further modulate 5‐HT release and regulate gastrointestinal peristalsis (Linan‐Rico et al. 2016). Research has also indicated a correlation between the purinergic signaling pathway and 5‐HT, with promising findings suggesting that the administration of TBB‐FA in FD rats led to alterations in metabolites concentrated in the purine signaling pathway. These observations offer promising prospects for the creation of innovative therapeutic agents and clinical strategies for FD management.

### 4.3 The Purinergic Signaling Pathway Presents a Novel Therapeutic Target for Addressing Gastrointestinal Disorders, Thereby Facilitating the Advancement of Innovative Drug Development for the Clinical Management of Functional Dyspepsia

The purinergic signaling pathway, a pivotal cellular communication conduit, is instrumental in orchestrating a myriad of physiological processes including cell growth, apoptosis, and inflammation. Recent research has highlighted the close association between the purinergic signaling pathway and the onset and progression of gastrointestinal disorders. Modulating this pathway has been shown to enhance the contractile function of gastrointestinal smooth muscle cells, expedite gastric emptying, and alleviate symptoms associated with FD (Inami, Kiyono, and Kurashima 2018). Consequently, targeting purinergic signaling and its receptors holds promise for the development of novel therapeutic agents for FD.

Current efforts in drug discovery targeting purinergic receptors have primarily concentrated on inflammatory bowel disease, with limited exploration of its potential in FD treatment. For instance, studies have demonstrated a positive correlation between the purine signaling receptor P2Y14R and inflammation severity in patients with ulcerative colitis (Liu et al. 2024). Additionally, male mice lacking P2Y14R exhibited reduced intestinal damage induced by sodium dextran sulfate. Building upon these findings, a classification strategy integrating virtual screening and chemical optimization led to the synthesis of a highly effective and low‐toxic small molecule P2Y14R antagonist, HDL‐16, which mitigated colitis induced by DSS through P2Y14R targeting. This discovery offers a promising therapeutic avenue for inflammatory bowel disease.

In line with the current research landscape and methodologies, preliminary investigations utilizing molecular docking technology were conducted to assess the interaction between naringin, neohesperidin, limonin, and nomilin‐four quality markers associated with the efficacy of “broad middle and distention” of FA‐P2Y1, a pivotal receptor in the purinergic signaling pathway. The docking analysis revealed strong binding affinities of these compounds with binding energies ranging from −7.63 to −7.79 kcal·mol^−1^, indicating their potential to interact with the P2Y1 receptor. This interaction may be crucial in promoting gastrointestinal smooth muscle cell contraction and alleviating gastrointestinal dysfunction. Consequently, future investigations by the research group will focus on leveraging the purinergic signaling pathway as a novel target for gastrointestinal motility disorders and identifying additional natural compounds with therapeutic potential for FD treatment.

## 5 Conclusion

The study introduced a novel technique for frying FA auxiliary resources by incorporating TBB. This approach seeks to integrate both medicinal and auxiliary components, aiming to enhance synergistic therapeutic effects and achieve high‐value applications. Based on the “intestinal flora‐SCFAs‐metabolomics” approach, the potential mechanisms underlying the therapeutic effect of frying FA with TBB on FD were explored. The feasibility of TBB frying as a WB stir‐bake substitute in FD therapy with FA was also evaluated in this study. The abundance of Bacteroides in the intestine of FD rats and the expression levels of propionate and isobutyrate after stir‐baking with TBB were found to have a high positive connection (*r* ≥ 0.7).

Accordingly, FA stir‐baked with TBB may improve the FD rats' intestinal flora, increase the number of Bacteroides, and subsequently increase the expression of SCFAs such as propionate and isobutyrate. Additionally, metabolomics analysis of the intestinal mucosa and serum showed significant differences in metabolites, mostly related to the “purine” metabolic signaling pathway. The processes of FA stir‐baked with TBB in FD therapy can be further investigated with a focus on the interaction of Bacteroides, SCFAs, and the purine pathway, thanks to these results. Moreover, the purinergic signaling system is a potential therapeutic target for gastrointestinal diseases, which will facilitate the development of new drugs for the clinical treatment of FD.

## Author Contributions

**Di Wang:** conceptualization (equal), project administration (supporting), writing – original draft (lead). **Xiaobo Zeng:** data curation (equal), investigation (lead), methodology (equal). **Xinge Wang:** conceptualization (equal), formal analysis (lead). **Wen Wen:** conceptualization (equal), methodology (equal). **Ping Wang:** data curation (lead), resources (supporting), software (lead), writing – review and editing (lead). **Shijun Xu:** funding acquisition (supporting), project administration (supporting), resources (equal), writing – review and editing (supporting).

## Conflicts of Interest

The authors declare no conflicts of interest.

## Data Availability

The data that support the findings of this study are available on request from the corresponding author.
